# 3D printable soft and solvent-free thermoplastic elastomer containing dangling bottlebrush chains†

**DOI:** 10.1039/d3ma00335c

**Published:** 2023-08-31

**Authors:** Vahid Asadi, Renee Dolleman, Jasper van der Gucht, Thomas E. Kodger

**Affiliations:** a Physical Chemistry and Soft Matter, Wageningen University & Research Stippeneng 4 6708 WE Wageningen The Netherlands thomas.kodger@wur.nl

## Abstract

Polymer networks containing bottlebrush chains are emerging materials with exceptionally soft and highly tunable mechanical properties. However, such materials have not been extensively implemented in functional processing techniques such as three-dimensional (3D) printing. Here, we introduce a new design of soft and solvent-free polydimethylsiloxane (PDMS)-based thermoplastic elastomer which contains dangling and space-filling bottlebrush chains, featuring a yield stress and a rapid recovery after stress removal; both required for high spatial fidelity 3D printing. The developed material is composed of two copolymers; the main building block is a diblock copolymer with linear polystyrene (PS) block and bottlebrush PDMS block (PS-*b*-bbPDMS) while the second component is PS-*b*-PDMS-*b*-PS triblock, self-assembling to a physical network. This design provides independent tunability of each structural parameter on the molecular level, hence, macroscopic control of the materials' mechanical properties. Multiple self-supportive 3D structures with spanning elements are 3D printed at elevated temperatures using a developed material with a low shear modulus of *G*′ = 3.3 kPa containing 3 : 1 molar ratio of diblock to triblock copolymers without the need for volatile solvent, or post-treatment. This 3D printing compatible design opens new opportunities to utilize the distinctive mechanical properties of bottlebrush materials for applications such as soft tissue scaffolds, sensors, actuators, and soft robots.

## Abstract 2

Designed super-soft and solvent-free bottlebrush thermoplastic elastomer compatible with 3D printing with tunability of each topological parameter at the molecular level.

## Graphical abstract

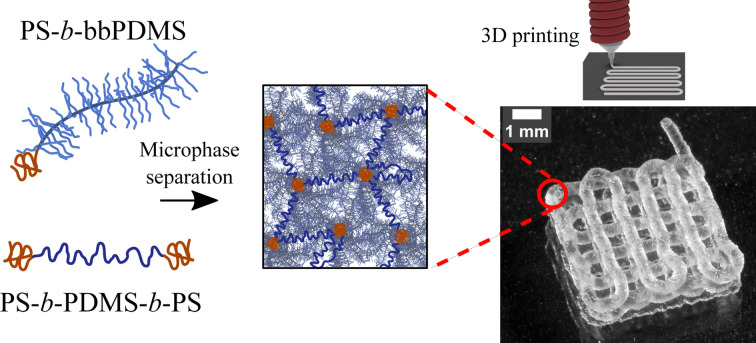

## 1 Introduction

Super-soft bottlebrush networks behaving as elastomers and thermoplastic elastomers are emerging classes of topologically-tunable materials derived from chemically or physically linked bottlebrush strands, respectively.^1–3^ Possessing exceptionally soft mechanical properties has made these materials appealing in applications such as biological tissue mimics,^4–6^ complex actuators,^7,8^ and capacitive pressure sensors.^9^ These materials exhibit substantially low shear moduli in the range of 1–100 kPa which cannot be achieved by conventional nonsolvated linear polymer networks due to inherent polymeric entanglements that impose a lower threshold in moduli.^10,11^ However, in the case of the bottlebrush topology, wherein a linear polymer backbone has been highly grafted with relatively short linear side chains, the inhibition of entanglements together with effective dilution imposed by grafted side chains renders a material with exceptionally soft mechanical properties without addition of any solvent or additives. In the past decade, the focus of the research was mainly to fundamentally understand the structure of bottlebrush chains and how the structural parameters affect the morphology and mechanics of bottlebrushes in the melt or in the network.^3,12–15^ These theoretical understanding of bottlebrush chains are continuously developing as, for instance, it has been recently reported that in bottlebrushes with an incompatible backbone and side chains, *i.e.* composites, the backbone folds into a cylindrical core to reduce the interfacial free energy.^16^ While bottlebrush soft materials offer huge potential tunability in their mechanical properties,^17–21^ they have been limited in use by a lack of knowledge into their response to conventional processing techniques such as molding and casting. Thus, developing soft materials that can be used with these, and more advanced processing methods, could open new avenues to utilize their distinctive mechanical properties in innovative ways.

The use of three-dimensional (3D) printing as a versatile processing technique is a prime example of how applications of bottlebrush elastomers can be expanded in various fields. This technique can create three-dimensional objects with intricate shapes, precise sizes, and composite compositions that are typically unachievable through traditional molding methods.^22–25^ Here, we focus on a variant of 3D printing known as direct ink writing (DIW) which is operationally simple, widely available, low-cost, and offers more freedom in material choice.^26,27^ In this technique, a yield-stress material is extruded through a narrow nozzle and experiences a phase transition causing solidification upon removing stress allowing the material to follow a predesigned spatial pattern. Two recently developed designs of bottlebrush soft materials have been explored for DIW printing.^28,29^ The first design involves highly asymmetric statistical bottlebrushes with two different side chains which self-assemble to form a well-ordered microstructure. This microstructure exhibits desirable yield stress behavior and allows for printing 3D structures.^28^ However, chemical crosslinking under UV illumination stabilizes the structure after printing which depends on the intensity of light and inevitably varies as light diffuses in the printed sample. This complexity increases the potential for uncontrolled crosslinking, which can affect the mechanical properties of the printed object.

Another approach uses linear-bottlebrush-linear (LBBL) triblock copolymers to create a physically-linked bottlebrush network, *i.e.* thermoplastic elastomer, which does not require a post-treatment crosslinking step.^5,30–32^ A PDMS-based LBBL thermoplastic elastomer operating at room temperature DIW printing in the way that the material is solvated with a volatile solvent that evaporates as the extruded material exits the nozzle, leading to solidification which provides enough mechanical strength for the printed feature.^29^ Although the solvent facilitates a sharp solid-to-liquid transition at lower stress values, its use presents challenges. Solvent evaporation can cause printed features to shrink and distort, and residual solvent may be harmful in certain applications, such as tissue engineering, when the material is in contact with living cells. Furthermore, the LBBL thermoplastic elastomer studied was synthesized with atom transfer radical polymerization, ATRP, using the “grafting through” approach, potentially resulting in inconsistent properties between batches due to complications such as termination and chain transfer events that lead to undesired impurities, including linear-bottlebrush diblocks (LBB) and bottlebrush homopolymers (BB).^33^ Quantitatively determining the composition of the bottlebrush diblock and triblock composition is not straightforward leading to inconsistency in the mechanical properties as the elasticity is predominantly dictated by the triblock constituent. Currently, there is no reported soft bottlebrush elastomeric material that can be directly 3D printed to create structures that are stable with time without the use of solvent or photocrosslinking, highlighting the need for alternative designs that accelerate the realization of these materials in additive manufacturing.

In this paper, we design a new class of soft and solvent-free PDMS-based thermoplastic elastomer which contains dangling and space-filling bottlebrush chains, featuring the shear stress yielding and fast recovery required for DIW. We show applicability of this material for 3D printing by shaping multiple 3D patterned structures which also possess spanning elements at elevated temperatures without any need for a supportive material or solvent or any post-treatment. The introduced two-component thermoplastic elastomer is composed of linear–bottlebrush (LB) diblock copolymer and linear ABA triblock compolymer. Bottlebrush diblock, as the main component, is synthesized through controlled anionic polymerization and the “grafting onto” approach,^34^ providing high control on the molecular structure and also independent tunability of each structural parameter, hence, tunability of materials mechanical properties. Feasibility of the LB synthesis, combined with commercially available ABA triblock copolymer which serves as a physical crosslinker, facilitate the adoption of this material for scientific research and can attract broad appeal in the context of additive manufacturing. We anticipate that this work will inspire further investigation into the design and potential applications of bottlebrush-based soft materials in various fields.

## 2 Experimental section

### Materials

All chemicals were used as received unless otherwise noted. *n*-Butyllithium (*n*-BuLi, 2 M in cyclohexane), *sec*-butyllithium (*sec*-BuLi, 1.4 M in cyclohexane), tetrahydrofuran (THF, anhydrous, ≥99.9%, inhibitor-free), styrene, platinum(0)-1,3-divinyl-1,1,3,3-tetramethyldisiloxane complex solution (Karstedts catalyst in xylene, Pt ≈ 2%), and chlorotrimethylsilane (purified by redistillation, ≥99%) were purchased from Sigma Aldrich. Hexamethylcyclotrisiloxane (D_3_, 95%), 1,3,5-trivinyl-1,3,5-trimethylcyclotrisiloxane (D_3_V), and chlorodimethylsilane (CDMS, 98%) were purchased from Gelest. 1,1,1,3,5,5,5-Heptamethyltrisiloxane (HMTS, ≥98%) was bought from TCI Chemicals. Linear polystyrene-*b*-polydimethylsiloxane-*b*-polystyrene (PS-*b*-PDMS-*b*-PS) triblock copolymers with two different molecular characteristics (P10730-SDMSS: 15-*b*-60-*b*-15 kg mol^−1^, *Đ* = 1.25 and P10782-SDMSS: 19.5-*b*-130-*b*-19.5 kg mol^−1^, *Đ* = 1.3) were purchased from Polymer Source, Inc. Anhydrous toluene was obtained from Acros Organics and other solvents such as cyclohexane, methanol, and acetone were purchased from Biosolve.

### Synthesis procedures

For anionic polymerizations, the glassware underwent overnight baking at 110 °C followed by immediate transfer to a nitrogen environment glove box. Unless otherwise specified, all reactions were conducted at room temperature. Purification of the D_3_ monomer was accomplished by sublimation on a Schlenk line. Cyclohexane was dried for at least 48 hours using activated molecular sieves (4 Angstrom, Sigma Aldrich) before use. Vacuum distillation at 40 °C was employed to purify styrene followed by storage at −80 °C in the presence of activated molecular sieves.

### Monohydride-terminated polydimethylsiloxane (PDMS-H)

*n*-BuLi (5.62 mL, 11.24 mmol) as the initiator was added to a solution of sublimed D_3_ (50.0 g, 224.76 mmol) in anhydrous cyclohexane (400.82 mL) inside the glove box. After an overnight reaction at room temperature, anhydrous THF (44.53 mL) as a promoter to ensure propagation was introduced to the reaction medium to achieve a solvent composition of cyclohexane/THF (90/10). The reaction continued for 20 hours until 50% conversion, determined by ^1^H NMR spectroscopy. Quenching of the reaction was accomplished by adding 2 molar equivalents of CDMS (2.5 mL, 22.47 mmol). The reaction mixture was then allowed to stand overnight, filtered, and the majority of the solvent evaporated by rotary evaporation. Finally, a strong vacuum with a vacuum trap was employed to remove any remaining unreacted monomers (D_3_) through sublimation and solvent.

### Synthesis of PS-*b*-PMVS diblock copolymer

*sec*-BuLi (322 μL, 0.45 mmol) was added to a solution of distilled styrene (4.7 g, 45.13 mmol) in anhydrous cyclohexane (151.17 mL) to initiate its polymerization inside the glove box. After 4 hours of complete polymerization, a stock solution of D_3_ (210 mg mL^−1^) was added to the reaction medium along with THF (1.52 mL) to change the nature of the end ion to silanolate ion, as discussed in our previous study.^34^ This ion change ensures simultaneous propagation of living chains, resulting in lower dispersity in the molecular weight. The reaction proceeded overnight, during which the orange color of the living polystyrene disappeared. To change the solvent composition to cyclohexane/THF (90/10), more THF (15.27 mL) was added, and then D_3_V (6.03 mL, 22.56 mmol) was quickly injected into the reaction flask. Polymerization of D_3_V continued for 24 hours and was quenched by chlorotrimethylsilane (115 μL, 0.9 mmol). The quenching step continued for another 24 hours followed by precipitation into methanol, and reprecipitation into methanol from a THF solution twice.

### Synthesis of PS-*b*-bbPDMS diblock copolymer

A relatively concentrated solution of 500 mg mL^−1^ was prepared by dissolving synthesized PDMS-H (14.03 g) and PS-*b*-PMVS (4.50 g) in 37.06 mL of anhydrous toluene under the fume hood. This mass ratio was selected by considering the molecular weights of PS-*b*-PMVS and PDMS-H (with 90% hydride end-functionality) to achieve 20% grafting density. An aliquot was taken as a reference point for further characterization, before adding 92 μL of Karstedts catalyst (2% platinum in xylene) to the reaction medium to achieve a catalyst concentration of 5 μL per gram of undissolved material. The temperature was increased to 80 °C to increase the rate of reaction, and the grafting reaction was monitored with ^1^H NMR spectroscopy. Additional PDMS-H (3.50 g) was added at later stages to reach higher grafting densities. Once the desired grafting density was achieved, excess HMTS (14.5 mL) was added to react with the remaining vinyl groups through the same hydrosilylation reaction. After solvent evaporation, the bottlebrush molecules were purified by dissolving them in a mixture of acetone/THF (80/20 v/v) and extracting the excess HMTS and unreacted PDMS-H chains *via* the addition of methanol (10 v% of solution) three times. The purified molecules were dried at 40 °C in a vacuum oven for 48 hours.

### Preparation of 3D printable thermoplastic elastomer

The purified PS-*b*-bbPDMS diblock and PS-*b*-PDMS-*b*-PS triblock copolymer were dissolved in THF with the diblock to triblock weight ratio of 7.11 (using 15-*b*-60-*b*-15 kg mol^−1^) and 3.78 (using 19.5-*b*-130-*b*-19.5 kg mol^−1^). These weight ratios were determind to reach a molar ratio of 3 : 1 for diblock to triblock based on their molecular weight. The solvent evaporated *via* a rotary evaporator and the obtained material was dried at 40 °C in vacuum oven for 48 hours.

### Molecular characterization

Size exclusion chromatography (SEC) experiments were conducted on an Agilent system equipped with a differential refractometer using one Agilent organic column (PLgel MIXED-D, 7.5 × 300 mm, 5 μm) with toluene or tetrahydrofuran as eluent at 35 °C and a flow rate of 1 mL min^−1^. Molecular weights and molecular weight distributions (*Đ*) were measured relative to linear polystyrene standards. Nuclear magnetic resonance (^1^H NMR) spectra were recorded by using Bruker 400 MHz instruments at 25 °C with deuterated chloroform (99.50% D).

### Rheology

Rheological experiments were performed on a stress-controlled rheometer (TA, Discovery Hybrid) with parallel plate geometry with a diameter of 8 mm. thermoplastic elastomer samples were formed as disks of 8 mm in diameter and thickness of 1 mm using a laboratory hot press at 120 °C under 2 bar pressure. After loading, the sample was squeezed to a gap size of slightly lower than 1 mm to establish good contact with the plate with a normal force of less than 0.1 N, followed by trimming the excess material. Oscillatory amplitude sweep measurements were done from 10^−2^ to 10^3^% strain and frequency of 1 Hz at 20 and 160 °C. The frequency sweep response of samples was captured from 10^−2^ to 10^1^ Hz with strain of 1% at 20 and 160 °C. To further probe the yielding transition, shear creep and recovery experiments with increasing step stress at both 20 and 160 °C were performed. Each cycle consisted of a creep time of 120 s and a recovery time of 240 s. In addition, oscillatory shear time sweeps with cyclic stresses were carried out where the strain amplitude changed between 0.1 (stress = 2.7 Pa < *τ*_*y*_ for 10 minutes) and 200 (stress = 1.6 kPa > *τ*_*y*_ for 12 seconds) for four consecutive cycles.

### Transmission electron microscopy (TEM)

Dark-field TEM images were captured using a JEM1400 microscope from JEOL, operating at 120 kV. Sample was prepared by depositing 10 μL of a 5 mg mL^−1^ solution in toluene onto 400 mesh formvar/carbon grids and incubated for two minutes without any negative staining. The prepared polymer solution was passed through a syringe filter with a pore size of 0.45 μm to remove possible dusts.

### Direct ink writing (DIW)

Direct-write printing was achieved by a custom-made setup equipped with a high-precision syringe pump (Chemyx, Nexus 3000) pushing the material out of a 2.5 mL gastight glass syringe while the printing platform moving in three dimensions. The glass syringe was equipped with a stainless steel tapered dispensing nozzle of inner diameter 0.4 mm (Nordson EFD, Gauge 22) and was wrapped by fiberglass heating rope, enabling injection at elevated temperatures. A temperature process controller (Omega, CN142) was used to adjust the temperature of the syringe to 160 °C by a feedback loop reading the temperature sensor and regulating the current in the heating rope. The print bed was a glass slide with the temperature of 20 °C. The motion system consisted of a motorized high-load vertical translation Stage (Thorlabs, MLJ150/M) for the Z direction and two linear motorized translation stages (Thorlabs, MTS50/M-Z8) covering the X and Y directions. The syringe pump and the motion system were controlled by a computer through a written MATLAB script. Pictures of the used setup are shown in Fig. S9 in the ESI.† Images of printed lines were captured with an Olympus BX60 polarizing optical microscope equipped with a DT70 color camera.

## 3 Results and discussion

### Molecular design and synthesis

The 3D printing of soft materials using extrusion-based 3D printing techniques requires materials that exhibit proper rheological behavior such as apparent viscosity, yield stress under shear and compression, and viscoelastic properties.^26,35^ A melt of homopolymer bottlebrush chains does not exhibit such a yield-stress behavior, yet are softer than polymers with linear chains of the same size due to the entanglement-free structure of the bottlebrush topology. To create the desired yield-stress behavior with required viscoelastic properties to facilitate 3D printing capability, bottlebrush chains have been prepared in the copolymerized state with blocks that undergo microphase separation into domains that from physical nodes in the bottlebrush network.^36^ Self-assembly of bottlebrush copolymers has been extensively studied, showing fast phase-separation kinetics and large-range structures, depending on the molecular architecture and most importantly the volume fraction of the minority block.^31,37,38^

**Fig. 1:**
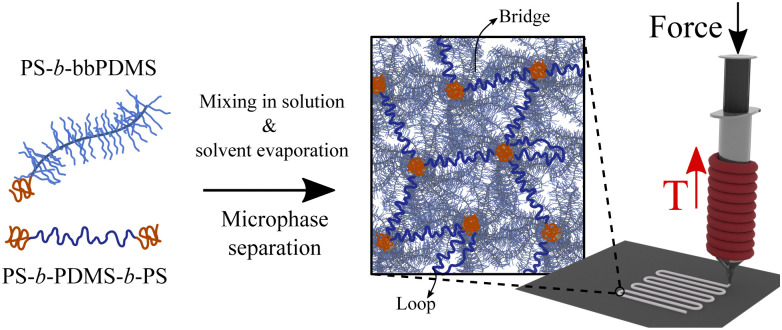
Design concept of soft and solvent-free bottlebrush thermoplastic elastomer for DIW printing at elevated temperature. PS-*b*-bbPDMS diblock and PS-*b*-PDMS-*b*-PS triblock copolymers are first separately dissolved in THF and then mixed in the solution state with a specific molar ratio, followed by solvent evaporation. As the solvent evaporates, the mixture self-assembles into microphase-separated domains connected by linear triblock copolymers, *i.e.* bridges, providing elasticity to the material. At elevated temperatures and under shear stress, the microphase-separated domains reversibly dissociate and rearrange, making it applicable for DIW printing.

This paper presents a new 3D printable soft material by leveraging microphase separation of block copolymers as the design principle. Our material is composed of two components allowing for a large design phase space, as illustrated in Fig. 1. This design enables precise control of material properties without the need for any additives or solvents, while also eliminating the need for additional post-treatment steps following the 3D printing process. The main component which occupies most of the sample volume is a diblock copolymer with linear PS block and bottlebrush PDMS block (PS-*b*-bbPDMS). The inherent incompatibility of the two blocks in these diblock chains causes them to self-assemble into anticipated micellar structures, creating microphase-separated domains in the melt state. This, in turn, introduces a yield-stress behavior. The second component is a linear ABA triblock copolymer with PS end blocks and PDMS middle block (PS-*b*-PDMS-*b*-PS) that bridges the microphase-separated domains, resulting in a physical network with elasticity, hence shape stability after being 3D printed. The majority of this physical network is composed of bottlebrush diblock chains which sterically-hinders the conformation of the stress-supporting behavior ABA triblock linker between PS domains. For this reason, we refer to the prepared network as a bottlebrush network even though the stress-supporting component in the network is the linear ABA triblock.

In this design framework, molecular structural parameters of the components and also their relative molar ratio collectively dictate the kinetics and morphology of the self-assembly, moduli, and yield stress behavior of the material. For the diblock copolymer, these topological parameters include the degrees of polymerization of linear PS block (*N*_L_), bottlebrush backbone (*N*_bb_), grafted side chains (*N*_sc_), and the grafting density (Gr), while for the linear ABA triblock copolymer, the structural parameters are degrees of polymerization of PS end blocks (*N*_A_) and PDMS middle block (*N*_B_). By altering these topological parameters, target elastic criteria for the bottlebrush diblock, *i.e.* PS-*b*-bbPDMS, can be achieved such that the material resists immediate flow under gravitational force but flows after some time. This target flow resistance in the bottlebrush diblock ensures that with the addition of only a small portion of ABA triblock, a self-supporting thermoplastic elastomer forms able to maintain its shape after being 3D printed. A preliminary investigation is done to determine the combination of the structural parameters for the bottlebrush diblock that meets this target: A library of PS-*b*-bbPDMS diblock samples was synthesized by changing the grafting density and degree of polymerization of side chains, keeping the total number of backbone monomers constant, see Fig. S1–S7 in the ESI.† A final topological criterion is that the length of the middle block in the ABA triblock be at least twice the bottlebrush backbone length (*N*_B_ ≥ 2*N*_bb_) to ensure the possibility of bridge formation between PS domains.

**Fig. 2:**
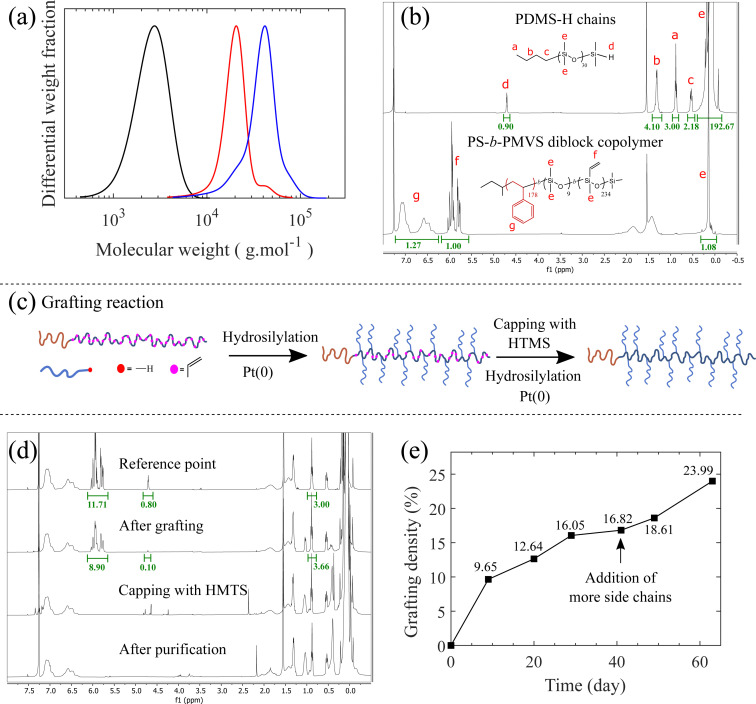
Molecular characterization of synthesized materials. (a) SEC curves for the precursors of the PS-*b*-bbPDMS diblock copolymer. PDMS-H side chains (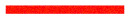) with *M*_n_ = 2260, *M*_w_ = 2700 g mol^−1^, and *Đ* = 1.19, PS block (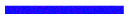) in the backbone with *M*_n_ = 18 600, *M*_w_ = 21 000 g mol^−1^, and *Đ* = 1.13, and PS-*b*-PMVS diblock (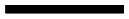) with *M*_n_ = 33 300, *M*_w_ = 40 800 g mol^−1^, and *Đ* = 1.22. (b) ^1^H NMR spectra of the PDMS-H (top: 400 MHz), CDCl_3_, 25 °C, *δ* = 0.07 ppm (m, 3H; –Si–CH_3_), 0.53 ppm (t, 2H, –CH_2_–), 0.88 ppm (t, 3H, –CH_3_), 1.30 ppm (m, 4H, –CH_2_=CH_2_), 4.70 ppm (m, 1H, –Si–H) and PS-*b*-PMVS diblock copolymer chains (bottom: *δ* = 0.07 ppm (m, 3H; –Si–CH_3_), *δ* = 5.72–6.10 ppm (m, 3H, –CH=CH_2_), *δ* = 6.25–7.25 ppm (m, 5H, –C_6_H_5_)). A short block of PDMS is incorporated between PS and PMVS for the synthesis reasons to change the nature of the PS end ion to silanolate ion that ensures simultaneous propagation of living chains, as described in our previous paper.^34^ (c) Schematic representation of the grafting reaction. (d) ^1^H NMR spectra for the grafting reaction. The –CH_3_ reference peak located at *δ* = 0.88 ppm has been adjusted due to the addition of more side chains during the grafting reaction. (e) Evolution of the grafting density calculated from the consumption of the vinyl groups on the backbone. 
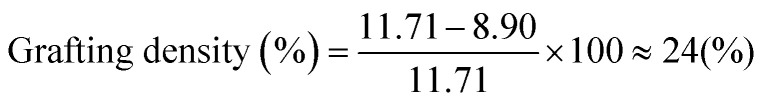
.

PS-*b*-bbPDMS diblock copolymer is synthesized in two main steps; first, the monohydride-terminated PDMS side chains (PDMS-H) and the linear PS-*b*-PMVS diblock copolymer are separately synthesized using living anionic polymerization. PDMS-H is synthesized through polymerization of D_3_ which initiated by *n*-BuLi and quenched with CDMS, leaving a hydride functional group at the end of each chain. To synthesize the linear PS-*b*-PMVS diblock copolymer, polymerization of styrene initiated by *sec*-BuLi, is continued by polymerization of D_3_V and quenched with CTMS, leaving a vinyl functional group on each repeating unit of the PMVS block. A more detailed synthesis discussion of these precursors have been reported in our previous paper.^34^ The molecular weight distributions and ^1^H NMR spectra of the synthesized PDMS-H side chain and PS-*b*-PMVS diblock copolymer are shown in Fig. 2a and b, indicating the formation of pure and low-dispersity polymer chains. The degree of polymerization of PDMS-H is 30 which is calculated by end group analysis from the ratio of –Si–CH_3_ (0.07 ppm) to –CH_3_ (0.88 ppm) signals in the ^1^H NMR spectrum which is also in agreement with the DP value derived from the SEC chromatogram. In addition, hydride end-functionalization is calculated from the ^1^H NMR spectrum to be around 90% for PDMS-H based on the ratio of –Si–H (4.70 ppm) to –CH_3_ (0.88 ppm) signals. In the case of the diblock copolymer, the DP of PS block is determined by the molecular weight values obtained from SEC for PS, while the DP of PMVS block is determined based on the ratio of the ring protons of styrene –C_6_H_5_ (6.25–7.25 ppm) and vinyl protons –CH=CH_2_ (5.72–6.10 ppm) which are 178 and 234, respectively, see ESI.†

In the second step, the PDMS-H side chains are linked to the PMVS block following the “grafting onto” approach by hydrosilylation reaction between silicone hydride and unsaturated vinyl groups in presence of a platinum (Karstedt's) catalyst, as shown schematically in Fig. 2c. As the grafting reaction continues, the amount of vinyl groups decreases and the reduction percentage of the vinyl groups provides the grafting density. By taking an aliquot at the beginning of the grafting reaction for ^1^H NMR measurement, the reduction of vinyls is quantified by considering the methyl –CH_3_ (*δ* = 0.88 ppm) group located on the other end of PDMS-H chains as the reference peak, which originates from the *n*-BuLi initiator, shown in Fig. 2d. This reference peak does not change during the grafting reaction and also is a clearly resolved peak that does not overlap with other peaks in the ^1^H NMR spectrum. The grafting density of the bottlebrush block can be tuned by changing the ratio of PDMS-H to linear backbone and monitored by ^1^H NMR spectroscopy, shown in Fig. 2e. The ratio of the side chains to backbone also affects the kinetics of the reaction; as the aimed grafting density can be achieved in shorter time by adding excess amount of side chains compared with the stoichiometric ratio. As grafting proceeds, the rate of reaction decreases due to the decreasing concentration of free chains and also the steric hindrance imposed by the grafted side chains. However, additional side chains can also be introduced to the reaction medium at any point to further increase the grafting density on the same batch, implying the controlability of the grafting step. After reaching the desired grafting density, 24% in this case, excess amount of HMTS is added to react with the remaining vinyl groups through the same hydrosilylation reaction which is proved by dissappearance of vinyl protons –CH=CH_2_ (5.72–6.10 ppm) in the ^1^H NMR spectrum in Fig. 2d. This capping of vinyl groups ensures thermal stability as vinyl groups can have undesirable covalent crosslinking reactions at higher temperature used during 3D printing.

The resulting PS-*b*-bbPDMS diblock copolymers are easily purified by precipitation as the molecular weight of bottlebrush chains, hence their solubility, differ significantly from the excess HMTS and ungrafted, relatively low molecular weight PDMS-H. The synthesized PS-*b*-bbPDMS diblock and PS-*b*-PDMS-*b*-PS triblock copolymer are dissolved in THF at a 3 : 1 molar ratio to ensure a homogeneous mixture of the two components. After dissolution, the solvent is evaporated leading to a physical network with microphase-separated domains as the potential 3D printable soft material.

### Rheology

**Fig. 3:**
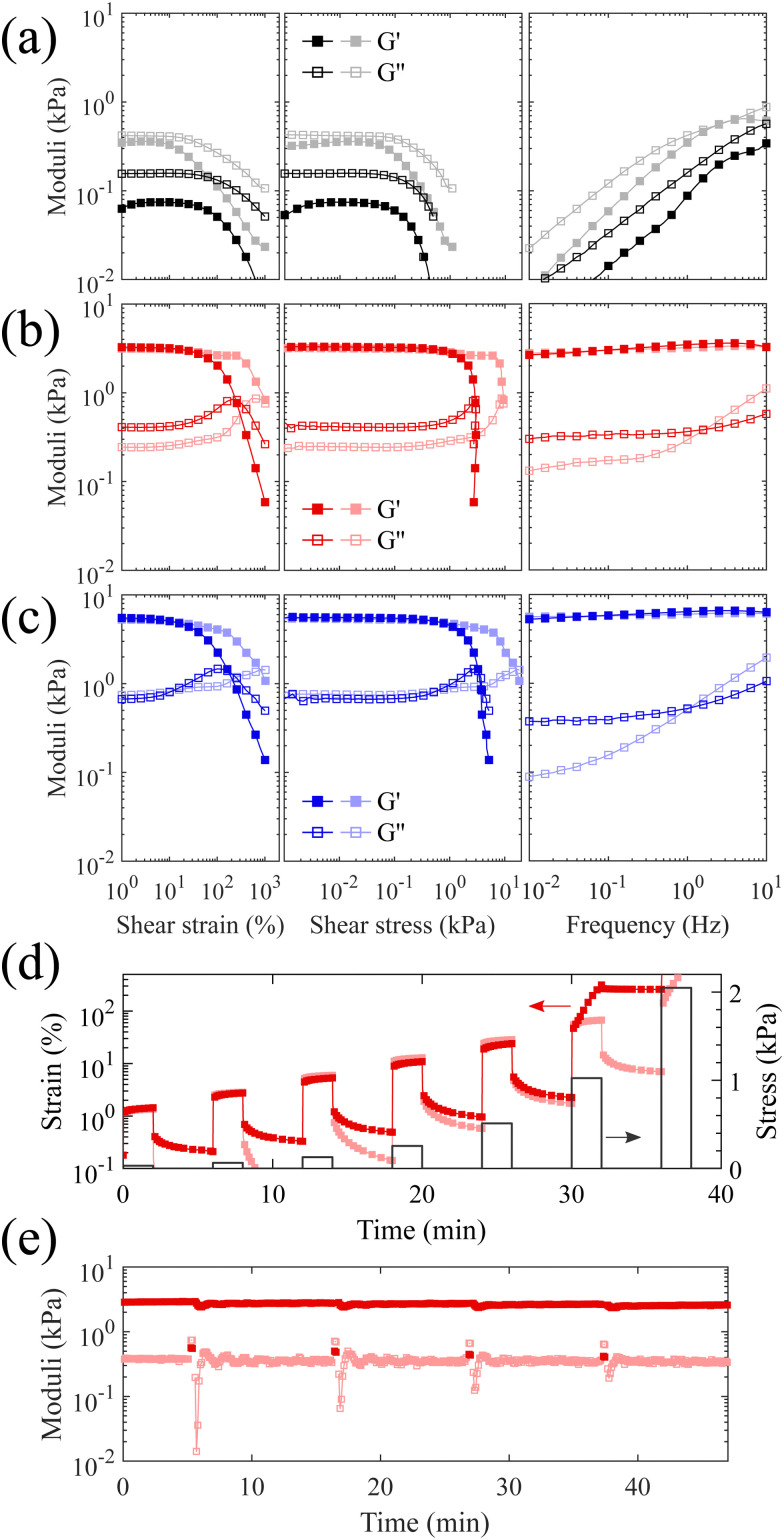
Rheological characterizations. Large amplitude oscillatory shear measurements at fixed frequency of 1 Hz and frequency sweep measurements at fixed strain of 1% at two different temperatures of 20 °C (light colors) and 160 °C (bright colors) for (a) the PS-*b*-bbPDMS diblock copolymer, (b) the mixture of PS-*b*-bbPDMS diblock and PS-*b*-PDMS-*b*-PS (15-*b*-60-*b*-15 kg mol^−1^) triblock with 3 : 1 molar ratio, and (c) the mixture of PS-*b*-bbPDMS diblock and PS-*b*-PDMS-*b*-PS (19.5-*b*-130-*b*-19.5 kg mol^−1^) triblock with 3 : 1 molar ratio. (d) Repeated shear creep and recovery experiments with increasing step stress at both 20 and 160 °C for the sample consisting of a mixture of PS-*b*-bbPDMS diblock and PS-*b*-PDMS-*b*-PS (15-*b*-60-*b*-15 kg mol^−1^) triblock with 3 : 1 molar ratio. (e) Oscillatory shear time sweeps with cyclic stresses where the strain amplitude changed between 0.1 (stress = 2.7 Pa < *τ*_*y*_) and 200 (stress = 1.6 kPa > *τ*_*y*_) for four consecutive cycles.

Two samples are prepared with the same 3 : 1 molar ratio of the synthesized PS-*b*-bbPDMS diblock with a grafting density of 24% in the bottlebrush block and PS-*b*-PDMS-*b*-PS triblock copolymer only differing in the length of triblock component. To evaluate the yielding behavior which is essential to 3D printing with DIW, the prepared materials are subjected to large amplitude oscillatory shear strain and frequency sweep measurements at both below (20 °C) and above (160 °C) glass transition temperature of the PS hard domains, shown in Fig. 3a–c. A sample consisting of only bottlebrush diblock, *i.e.* PS-*b*-bbPDMS diblock copolymer, shows liquid-like behaviour where the storage modulus is always lower than the loss modulus at both temperatures and all frequencies, shown in Fig. 3a. This sample becomes softer by heating where the storage modulus at low strains decreases from 360 Pa at 20 °C to 75 Pa at 160 °C when oscillating at 1 Hz. The value of the storage modulus in this sample is also dependent on the frequency, covering almost two orders of magnitude, as at low frequencies the sample has enough time to relax by structural rearrangements. This sample also features stress yielding behavior, experiencing a sudden drop in storage modulus with onset point around 90 and 105 Pa shear stress at 20 °C and 160 °C, respectively. The yielding behavior of bottlebrush diblock even in the absence of any bridging chains is due to the jamming of the microphase-separated micellar structures in the melt state, leading to a low yield stress value because of the structural rearrangement under stress. The yield stress at 160 °C is slightly higher compared to 20 °C, with a negligible difference of approximately ∼15 Pa. This difference could be attributed to an error in defining the onset point where the modulus starts to decline, or to the possibility of incomplete capping of vinyl groups with HMTS. Although there are no corresponding signals about remaining vinyls after the capping step in the ^1^H NMR spectrum, even a few occurrences of crosslinking can cause some change especially in such a super-soft sample but becomes unnoticeable in stiffer samples. This sample is not 3D printable by its own and flows under its own weight after being 3D printed due to the liquid-like behavior.

**Fig. 4:**
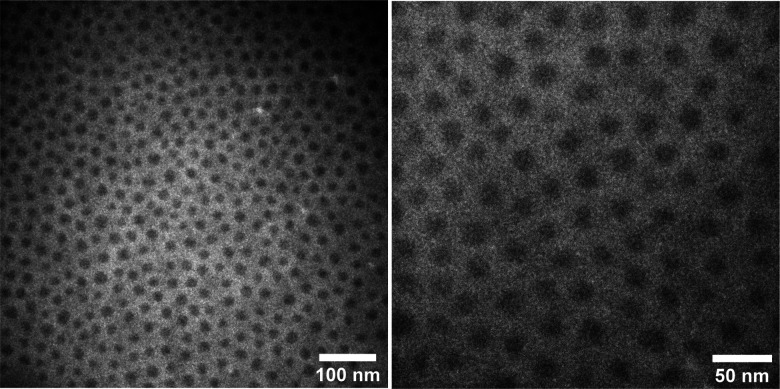
Dark-field TEM images of two different magnification for the mixture of synthesized PS-*b*-bbPDMS diblock and PS-*b*-PDMS-*b*-PS (15-*b*-60-*b*-15 kg mol^−1^) triblock with 3 : 1 molar ratio. The dark spherical features are domains formed by PS end blocks, whereas the white region is the matrix of PDMS.

The addition of PS-*b*-PDMS-*b*-PS triblock chains ensures interconnection of microphase-separated domains, leading to an elastic network with solid-like behavior. The contour length of the added linear ABA triblock should be sufficient to span two different PS domains. To this end, knowledge of the phase-separated morphology, *i.e.* domain size and domain distance, leads to logical selection on the molecular weight of ABA triblock needed to ensure interdomain connection. The volume fraction of PS in the prepared thermoplastic elastomers containing short triblock chains (15-*b*-60-*b*-15 kg mol^−1^) is around 15%, which likely leads to phase separated domains when in equilibrium based on a morphological study reported for an analogous PDMS-based LBBL material.^31^ To verify the anticipated morphology, dark-field TEM images of this sample are shown in Fig. 4 which are in excellent agreement with the reported study,^31^ showing regularly-spaced PS domains perpendicular to the substrate. The distribution of the PS domain size is shown in Fig. S8 in the ESI,† with the average value of 16.1 ± 3.3 nm.

However, it is likely that the samples do not uniformly contain this equilibrium morphology, but are trapped in a non-equilibrium state because of the fast solvent evaporation in the preparation step and also high shear during 3D printing. Therefore, the phase-separated morphology of samples is referred to as the micellar PS domains, which may be spherical or cylinderical or even disordered, linked by ABA triblock copolymer as evidenced in Fig. 3b and c where the storage modulus is one order of magnitude higher than the loss modulus. The storage modulus at low strains increases to 3.3 kPa for the sample containing short triblock chains (15-*b*-60-*b*-15 kg mol^−1^) and 5.6 kPa in the case of long triblock chains (19.5-*b*-130-*b*-19.5 kg mol^−1^); exceedingly low values in modulus for a melt are likely due to the entanglement-free bottlebrush topology. In addition, for strains lower than ∼10% the storage modulus is almost frequency and temperature independent for both prepared samples, meaning that by only heating at low strains the PS end blocks of the triblock chains cannot be pulled out of the PS hard domains and the network is reminiscent of a perfect rubber. However, increasing the temperature from 20 to 160 °C clearly affects the yield stress, reducing it from 7 to 1 kPa for the sample with short triblock chains and 6 to 0.8 kPa for the sample with long triblock chains. This decrease in yield stress at higher temperatures improves the flow inside the nozzle during 3D printing and prevents the accumulation of internal stress inside the sample, which could cause deformation after 3D printing. Both samples exhibit nearly the same yielding behavior, regardless the length of the triblock chains, *i.e.* the length of the bridges between PS domains. This is because the yielding in the system is most probably caused by the PS end blocks being pulled out of the PS hard domains, which results in structural rearrangement. The stiffness of the samples, however, is dependent on the length of the triblock chains, with the sample containing longer triblocks exhibiting a higher storage modulus. This may be attributed to the higher number density of bridges between the microphase-separated PS domains, resulting from a greater likelihood of bridge formation between distinct PS domains, rather than loops. The temperature dependency of the yield stress is advantageous from 3D printing point of view as it allows shear-induced yielding of the material during the injection at higher temperatures, while the material becomes stable when stress is released after the injection. Moreover, the proposed system allows tuning of the modulus by changing the length of linear ABA triblock and its molar ratio to bottlebrush diblock, which increases the applicability of these materials in different applications. Focusing on the softer modulus sample, to further investigate the yield stress behavior, a multiple-step creep test is also performed starting with a low stress which doubles in each subsequent step, shown in Fig. 3d. The sample demonstrates plastic deformation at 1 kPa stress at 160 °C which is fully consistent with the yield stress value obtained from the oscillatory stress sweep.

The solid-to-liquid transition of the bottlebrush thermoplastic elastomer under shear at high temperatures is highly reversible and recovers fast. This is evidenced by the recovery experiment, shown in Fig. 3e, where the sample repeatedly oscillates between two applied stresses which are smaller and higher than the measured yield stress. The storage modulus recovers to its initial value immediately after releasing the stress during the measured four cycles. This recovery in mechanical properties validates that the yielding of the bottlebrush thermoplastic elastomer is actually due to the rearrangements of the triblock chains by being pulled out from one domain and entering into another domain. Therefore, the speculated chain scission under shear is not likely for the bridging triblock chains since the broken chains are not able to reform the bridges which would lead to a lower modulus.

### 3D printing of bottlebrush-containing thermoplastic elastomer

**Fig. 5:**
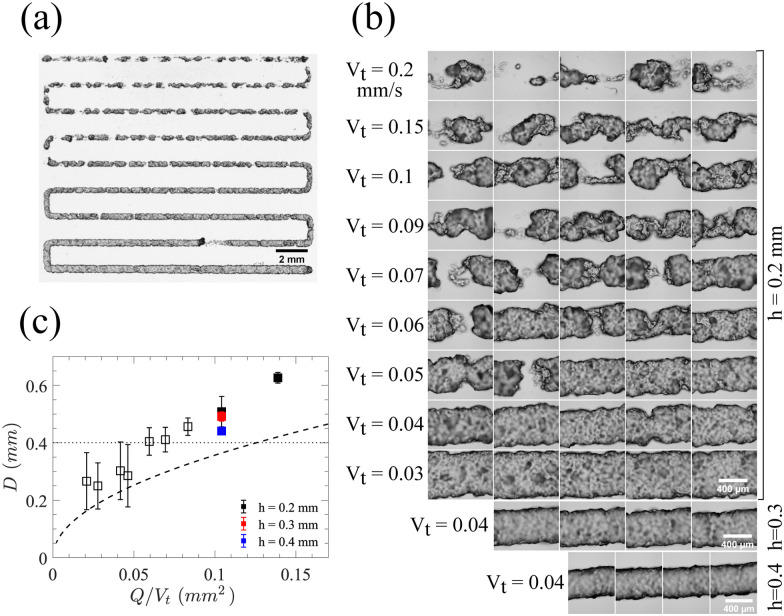
Control on the quality of 3D printed lines. (a) Zoom out view of 3D printed lines through 0.4 mm nozzle by changing the print speed while keeping the flow rate and layer thickness constant. (b) Optical microscopy images captured in multiple positions along the 3D printed lines. The bottom two images correspond to lines printed at the same print speed and flow rate but different layer thickness. (c) Dependence of line width on mentioned printing parameters. Solid symbols represent continuous lines while open symbols represent lines that break during printing. Dashed line shows the predicted diameter of a circular line based on conservation of volume, while the dotted horizontal line indicates the nozzle inner diameter. Lines have higher width than predicted due to the die swell and an oblate cross-section.

To demonstrate the 3D printability of the prepared bottlebrush thermoplastic elastomer, various features are 3D printed and examined to meet specific requirements for high-fidelity printing. The first and most crucial requirement is the ability to print stable and continuous lines. Using the softer thermoplastic elastomer for 3D printing experiments, *i.e.* the mixture of PS-*b*-bbPDMS diblock and PS-*b*-PDMS-*b*-PS (15-*b*-60-*b*-15 kg mol^−1^) triblock copolymer with 3 : 1 molar ratio, continuous lines are deposited at 160 °C through a nozzle with an inner diameter of 0.4 mm. This sample is chosen for 3D printing experiments since it shows a slightly faster transition at the yield point from solid-like to liquid-like behavior, as evidenced by a steeper drop of the modulus in Fig. 3b and c. However, since both samples have almost the same yield stress behavior at 160 °C, we expect similar 3D printing quality for the other sample as well. These 20 mm-long printed lines are deposited by tuning the nozzle translational speed (*V*_t_) and the distance between the nozzle tip and surface (*h*), while maintaining a constant flow rate (*Q*), shown in Fig. 5a with different *V*_t_ for each printed line, ranging from 0.2 (top line) to 0.03 mm s^−1^ (bottom line), with a constant *Q* of 15 μL h^−1^ and *h* of 0.2 mm (Movie S1, ESI†). As *V*_t_ decreases, fewer breakage points are observed along the printed lines until forming a continuous line. To investigate the effect of printing parameters on the width (*D*) of printed lines, lines are viewed under an optical microscope at points along the lines, as shown in Fig. 5b with the corresponding widths as a function of *Q*/*V*_t_ shown in Fig. 5c.

At low local injection rates, defined as *Q*/*V*_t_, the printed lines break due to the elasticity of the bottlebrush material when the stress is removed rather than plastic deformation which would lead to thinner lines. However, at a certain local injection rate the printed lines become continuous since enough material is being injected to fill the space of the printing trajectory. The width of lines also depends on the distance of the nozzle tip from the surface (*h*), which affects the extent to which the printed line is pressed onto the surface. By increasing *h*, the width of the line reduces due to a lower contact with the surface, as shown in Fig. 5b and c. The width of a continuous line is then dictated by the nozzle inner diameter and the degree of die swell which is found to be 13% (Fig. S10 in ESI†). For thinner lines, a smaller nozzle inner diameter is required. As printing proceeds at a constant *Q* and the bottlebrush thermoplastic elastomer does not involve any volume change, driven by solvent evaporation or UV crosslinking, the line's diameter can be predicted from the conservation of volume and assuming a circular cross-section using 
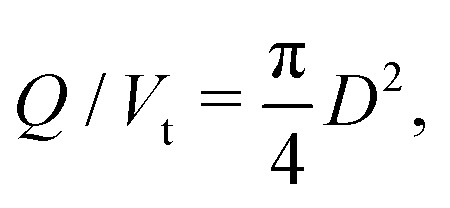
 as shown by the dashed line in Fig. 5c. Although the printed lines are known to deviate slightly from a circular cross-section, the theoretical prediction provides a narrow range of printing parameters indicating optimal 3D printing quality.

**Fig. 6:**
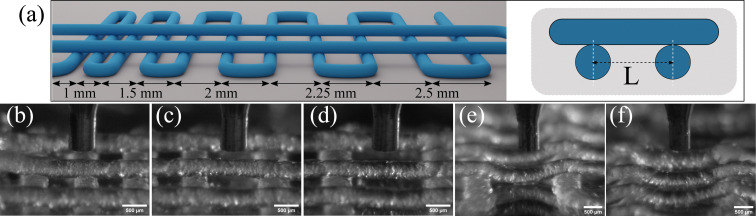
3D printing of a two-layer log-pile structure with systematically increasing the center-to-center distance between lines (*L*). (a) Schematic representation of the designed experiment, showing the selected *L* to be explored. Captured images of 3D printed lines with *L* distance of (b) 1 mm, (c) 1.5 mm (d) 2 mm, (e) 2.25 mm, and (f) 2.5 mm.

While a continuous printed line is important, moving in the third dimension requires free-standing structures that either span a small gap, or support the weight of the lines above, *i.e.* self-supporting. A 3D printed two-layer log-pile structure, as sketched in Fig. 6a, is a commonly used prototypical structure for 3D printability studies of materials using the DIW method, which produces a porous 3D structure. In such a test, the center-to-center distance between lines, defined as *L*, systematically increases from 1 to 2.5 mm. The ability of the lines on the upper layer to be laid on the lower-layer lines without sagging is critical to achieve high 3D print spatial fidelity.

To investigate the maximum *L* at which the bottlebrush thermoplastic elastomer material can be 3D printed, a first layer is printed with several gap lengths using *Q* = 18 μL h^−1^, *V*_t_ = 0.04 mm s^−1^, and *h* = 0.3 mm; these printing parameters ensure continuous lines are slightly pressed to the substrate for a good and stable contact. All the printing parameters are summarized in Table S1 (ESI†). Subsequently, the second layer is printed with *Q* = 15 μL h^−1^, *V*_t_ = 0.04 mm s^−1^ and a *h* = 0.4 mm to ensure that these lines are only laid on the first-layer lines and not pushed into the free space between lines (Movie S2, ESI†), as shown in Fig. 6b–f. The second-layer lines are consistently self-supportive for *L* up to 2 mm, however, the lines are sagging and touching the substrate as the line distance reaches *L* ≥ 2.25 mm. Gravity-induced deflection of an end-supported beam with circular cross section can be used for prediction of the maximum spanning length (*L*_0_ = *L* − *D*) for spanning lines to be self-supportive. A study based on this elastic beam theory on the shape evolution of the spanning elements in colloidal inks shows that the minimum modulus required to have a vertical deflection of less than 5% of the line diameter (*D*) at a span length (*L*_0_) is *G*′ ≥ 1.4*γ*(*L*_0_/*D*)^4^*D*, where *γ* is the specific weight of the material^39^. Using this bending mode approximation and the known modulus of the 3D printed material, *G*′ = 3.3 kPa, *L*_0_ ∼ 2 mm is in a good agreement with the observations in Fig. 6; at *L* ∼ 2.25 mm, the beam is no longer self-supportive and contacts the substrate, as seen in Fig. 6e. As the printing nozzle traverses the gap, sagging is also observed for smaller *L*, but the printing line recovers to a straight line when it reaches the next supporting line before contacting the substrate, as can be seen in Movie S2 (ESI†). Therefore, the maximum spanning distance which ensures a self-supporting structure can also be tuned by the thickness of the first layer, *i.e.* increasing the free space under the lines before reaching the substrate.

**Fig. 7:**
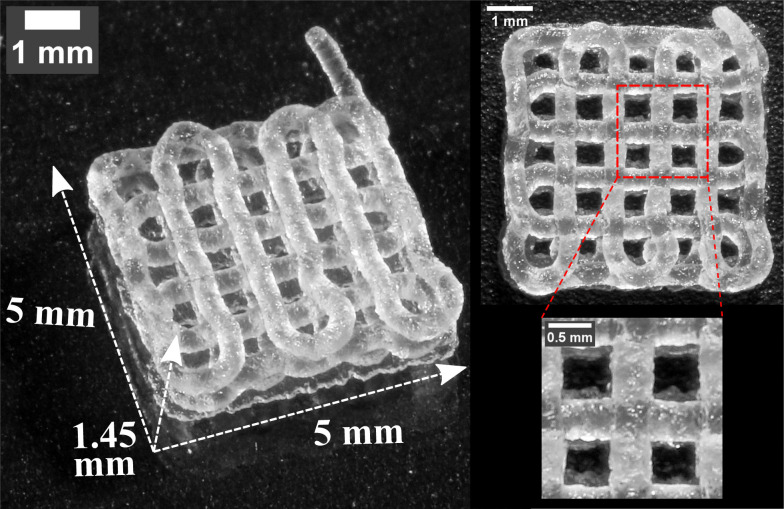
A log-pile structure composed of 4 layers (5 mm by 5 mm by 1.45 mm) 3D printed with *L* = 1 mm. Top-down perspective and the magnified view are shown on the right side of the picture.

Subsequently, with these printing parameters *Q*/*V*_t_, *h*, *L*, a full 3D, porous, four layer, log-pile structure is 3D printed (Movie S3, ESI†) to demonstrate the potential usage of the thermoplastic elastomer for complex 3D structures, as shown in Fig. 7. The structure is printed with *Q*/*V*_t_ = 0.125 mm^2^, *L* = 1 mm, and with *h* = 0.25 mm for the first layer and with *h* = 0.4 mm for the remaining layers. From a top-down perspective, the printed lines on different layers align with each other and form void spaces in a square shape, resulting in excellent positional shape fidelity. However, at the perimeter of the structure where there is a turn in the printer nozzle trajectory, the resulting lines do not position themselves as expected. This discrepancy between the designed path and the actual positioning of the lines at the perimeter is due to a force imbalance where in the center of the structure, each soft printed line is held in place symmetrically by force contact with neighboring lines; by contrast, on the perimeter this neighbor contacts are asymmetric. Importantly, as the scale of a 3D printed structure increases beyond this limited 5 mm by 5 mm pattern, this perimeter mismatch becomes an insignificant volume percentage of the total structure.

## 4 Conclusions

We have developed a new class of 3D print compatible soft and solvent-free PDMS-based thermoplastic elastomer which contains dangling and space-filling bottlebrush chains, featuring shear stress yielding and fast recovery, both required for 3D printing. This thermoplastic elastomer is made up of two components; a PS-*b*-bbPDMS bottlebrush diblock, as the bulk component which self-assembles into micro-phase separated domains with micellar structure, which are bridged together by linear PS-*b*-PDMS-*b*-PS, as the second component. This design enables a material with shear-induced yielding at temperatures higher than *T*_g_ of PS during the injection and fast modulus recovery after stress release, manifested by the recovery experiment by repeatedly oscillating at stresses lower and higher than the measured yield stress. 3D printability of the designed material is realized by first printing continuous lines on a surface through optimizing the 3D printing processing parameters. A showcase material with a low shear modulus of *G*′ = 3.3 kPa and yield stress of ∼1 kPa at 160 °C is used for 3D printing, containing 3 : 1 molar ratio of diblock to triblock copolymers in which PS-*b*-bbPDMS bottlebrush diblock has a grafting density of 24% with side chain DP of 30. Subsequently, continuous lines are shaped into a self-supportive 3D porous log-pile structure composed of multiple layers with center-to-center distance (*L*) of 1 mm between 3D printed lines, resulting in an excellent positional shape fidelity. In addition, it is shown that the printed lines are able to span over a longer distance up to *L* = 2 mm, studied by 3D printing of a two-layer log-pile structure while *L* was systematically increased. Although the main novelty of this work stands in the aspect of material development, few preliminary 3D printing experiments is performed which highlight the potential usage of the introduced soft thermoplastic elastomer for further in-depth post-printing analysis and also the fabrication of complex 3D structures. From the synthesis point of view, the introduced design ensures independent control and tunability of each structural parameter on the molecular level, hence, provides a design space to control macroscopic mechanical properties of the materials for specific application. Moreover, using anionic polymerization for synthesis, an industrial method producing yearly kilograms of polymers, possibly enables feasible scale-up and mass production as the necessary industrial equipment and expertise already exist. We envision that the introduced material, featuring a straightforward synthesis route and being compatible with widely-available DIW printing technique which produces self-supportive 3D structures without the need for any solvent or post-treatment, will attract broad interest in the materials science community. Thus, it may facilitate the implementation of bottlebrush soft materials in innovative applications where 3D patterning of the used material is required, such as soft tissue scaffolds,^40^ sensors,^41^ actuators,^42^ and soft robots.^43^

## Conflicts of interest

There are no conflicts to declare.

## Supplementary Material

MA-004-D3MA00335C-s001

MA-004-D3MA00335C-s002

MA-004-D3MA00335C-s003

MA-004-D3MA00335C-s004

## References

[cit1] Verduzco R., Li X., Pesek S. L., Stein G. E. (2015). Chem. Soc. Rev..

[cit2] Xie G., Martinez M. R., Olszewski M., Sheiko S. S., Matyjaszewski K. (2019). Biomacromolecules.

[cit3] Pan T., Dutta S., Kamble Y., Patel B. B., Wade M. A., Rogers S. A., Diao Y., Guironnet D., Sing C. E. (2022). Chem. Mater..

[cit4] Vatankhah-Varnosfaderani M., Daniel W. F. M., Everhart M. H., Pandya A. A., Liang H., Matyjaszewski K., Dobrynin A. V., Sheiko S. S. (2017). Nature.

[cit5] Keith A. N., Vatankhah-Varnosfaderani M., Clair C., Fahimipour F., Dashtimoghadam E., Lallam A., Sztucki M., Ivanov D. A., Liang H., Dobrynin A. V., Sheiko S. S. (2020). ACS Cent. Sci..

[cit6] Kostrov S. A., Dashtimoghadam E., Keith A. N., Sheiko S. S., Kramarenko E. Y. (2021). ACS Appl. Mater. Interfaces.

[cit7] Vatankhah-Varnoosfaderani M., Daniel W. F. M., Zhushma A. P., Li Q., Morgan B. J., Matyjaszewski K., Armstrong D. P., Spontak R. J., Dobrynin A. V., Sheiko S. S. (2017). Adv. Mater..

[cit8] Karimkhani V., Vatankhah-Varnosfaderani M., Keith A. N., Dashtimoghadam E., Morgan B. J., Jacobs M., Dobrynin A. V., Sheiko S. S. (2020). ACS Appl. Polym. Mater..

[cit9] Reynolds V. G., Mukherjee S., Xie R., Levi A. E., Atassi A., Uchiyama T., Wang H., Chabinyc M. L., Bates C. M. (2020). Mater. Horiz..

[cit10] Daniel W. F. M., Burdyńska J., Vatankhah-Varnoosfaderani M., Matyjaszewski K., Paturej J., Rubinstein M., Dobrynin A. V., Sheiko S. S. (2016). Nat. Mater..

[cit11] Cai L.-H., Kodger T. E., Guerra R. E., Pegoraro A. F., Rubinstein M., Weitz D. A. (2015). Adv. Mater..

[cit12] Zhulina E. B., Sheiko S. S., Borisov O. V. (2022). Soft Matter.

[cit13] Liang H., Cao Z., Wang Z., Sheiko S. S., Dobrynin A. V. (2017). Macromolecules.

[cit14] Liang H., Sheiko S. S., Dobrynin A. V. (2018). Macromolecules.

[cit15] Liang H., Wang Z., Sheiko S. S., Dobrynin A. V. (2019). Macromolecules.

[cit16] Nian S., Huang B., Freychet G., Zhernenkov M., Cai L.-H. (2023). Macromolecules.

[cit17] Sheiko S. S., Dobrynin A. V. (2019). Macromolecules.

[cit18] Self J. L., Sample C. S., Levi A. E., Li K., Xie R., De Alaniz J. R., Bates C. M. (2020). J. Am. Chem. Soc..

[cit19] Keith A. N., Clair C., Lallam A., Bersenev E. A., Ivanov D. A., Tian Y., Dobrynin A. V., Sheiko S. S. (2020). Macromolecules.

[cit20] Dashtimoghadam E., Fahimipour F., Keith A. N., Vashahi F., Popryadukhin P., Vatankhah-Varnosfaderani M., Sheiko S. S. (2021). Nat. Commun..

[cit21] Maw M., Morgan B. J., Dashtimoghadam E., Tian Y., Bersenev E. A., Maryasevskaya A. V., Ivanov D. A., Matyjaszewski K., Dobrynin A. V., Sheiko S. S. (2022). Macromolecules.

[cit22] Truby R. L., Lewis J. A. (2016). Nature.

[cit23] Wallin T. J., Pikul J., Shepherd R. F. (2018). Nat. Rev. Mater..

[cit24] Yuk H., Lu B., Lin S., Qu K., Xu J., Luo J., Zhao X. (2020). Nat. Commun..

[cit25] Choi C., Okayama Y., Morris P. T., Robinson L. L., Gerst M., Speros J. C., Hawker C. J., Read de Alaniz J., Bates C. M. (2022). Adv. Funct. Mater..

[cit26] Saadi M. A. S. R., Maguire A., Pottackal N. T., Thakur M. S. H., Ikram M. M., Hart A. J., Ajayan P. M., Rahman M. M. (2022). Adv. Mater..

[cit27] Tang M., Zhong Z., Ke C. (2023). Chem. Soc. Rev..

[cit28] Xie R., Mukherjee S., Levi A. E., Reynolds V. G., Wang H., Chabinyc M. L., Bates C. M. (2020). Sci. Adv..

[cit29] Nian S., Zhu J., Zhang H., Gong Z., Freychet G., Zhernenkov M., Xu B., Cai L.-H. (2021). Chem. Mater..

[cit30] Nian S., Lian H., Gong Z., Zhernenkov M., Qin J., Cai L.-H. (2019). ACS Macro Lett..

[cit31] Nian S., Fan Z., Freychet G., Zhernenkov M., Redemann S., Cai L.-H. (2021). Macromolecules.

[cit32] Nian S., Cai L.-H. (2022). Macromolecules.

[cit33] Cong Y., Vatankhah-Varnosfaderani M., Karimkhani V., Keith A. N., Leibfarth F. A., Martinez M. R., Matyjaszewski K., Sheiko S. S. (2020). Macromolecules.

[cit34] Asadi V., Li X., Ruggeri F. S., Zuilhof H., van der Gucht J., Kodger T. E. (2022). Polym. Chem..

[cit35] Jiang T., Munguia-Lopez J. G., Flores-Torres S., Kort-Mascort J., Kinsella J. M. (2019). Appl. Phys. Rev..

[cit36] Xie R., Mukherjee S., Levi A. E., Self J. L., Wang H., Chabinyc M. L., Bates C. M. (2021). Macromolecules.

[cit37] Fei H.-F., Yavitt B. M., Hu X., Kopanati G., Ribbe A., Watkins J. J. (2019). Macromolecules.

[cit38] Liberman L., Coughlin M. L., Weigand S., Edmund J., Bates F. S., Lodge T. P. (2022). Macromolecules.

[cit39] Smay J. E., Cesarano J., Lewis J. A. (2002). Langmuir.

[cit40] Guo J., Li Y., Gao Z., Lyu J., Liu W., Duan Y., Zhou L., Gu Q. (2022). J. Cell. Physiol..

[cit41] Guo S.-Z., Qiu K., Meng F., Park S. H., McAlpine M. C. (2017). Adv. Mater..

[cit42] Schaffner M., Faber J. A., Pianegonda L., Rühs P. A., Coulter F., Studart A. R. (2018). Nat. Commun..

[cit43] Kim Y., Yuk H., Zhao R., Chester S. A., Zhao X. (2018). Nature.

