# Supplementary material for: 3D printable soft and solvent-free thermoplastic elastomer containing dangling bottlebrush chains
Source: Mater Adv. 2023 Aug 31;4(22):5535–45. doi: 10.1039/d3ma00335c (PMC10642180; doi:10.1039/d3ma00335c)
Supplement: MA-004-D3MA00335C-s004 [file MA-004-D3MA00335C-s004.pdf]

Electronic Supplementary Material (ESI) for Materials Advances.
This journal is © The Royal Society of Chemistry 2023

Supporting Information:

3D Printable Soft and Solvent-free Thermoplastic Elastomer Containing Dangling Bottlebrush Chains

Vahid Asadi , Renee Dolleman , Jasper van der Gucht , and Thomas E. Kodger*

Physical Chemistry and Soft Matter, Wageningen University & Research, Stippeneng 4, Wageningen 6708 WE, The Netherlands, Email Address: thomas.kodger@wur.nl

The PDF file includes:

- Molecular characterization of the synthesized PS-b-bbPDMS diblock
- Figure S1: Schematic representation of the structural parameters of PS-b-bbPDMS diblock.
- Figure S2: ^{1}H NMR spectra of the PDMS-H chains with different degree of polymerization.
- Figure S3: Molecular characterization of preliminary synthesized PS-*b*-PMVS diblock copolymer.
- Figure S4: ^{1}H NMR spectra for synthesis of the PS-*b*-bbPDMS diblock copolymer with different length of grafted side chains and 10% grafting density.
- Figure S5: ^{1}H NMR spectra for synthesis of the PS-*b*-bbPDMS diblock copolymer with different length of grafted side chains and 20% grafting density.
- Figure S6: ^{1}H NMR spectra for synthesis of the PS-*b*-bbPDMS diblock copolymer with different length of grafted side chains and 30% grafting density.
- Figure S7: Gravitational flow experiment.
- Figure S8: PS domain size distribution obtained from TEM image.
- Figure S9: Custom-made 3D printing setup used for DIW printing of bottlebrush thermoplastic elastomer.
- Figure S10: Calculation of the percentage of die swell.
- Table S1: Overview of the 3D printing parameters.

Other Supplementary Material for this manuscript includes the following:

- **Movie S1 (.avi format, duration: 00:01:17):** Printed lines deposited with a constant Q of 15 $\mu L/hr$ and h of 0.2 mm but varied V_t ranging from 0.2 to 0.03 mm/s. The movie is 40 times real-time.
- **Movie S2 (.avi format, duration: 00:00:44):** Two-layer log-pile structure with L ranging from 1 to 2.5 mm. The movie is 40 times real-time.
- **Movie S3 (.avi format, duration: 00:00:35):** log-pile structure composed of four layers 3D printed with Q/V_t = 0.125 mm^2, L = 1 mm, and with h = 0.25 mm for the first layer and with h = 0.4mm for the remaining layers. The movie is 100 times real-time.

1 Molecular characterization of PS-*b*-bbPDMS diblock

Calculation of DP of PMVS block:

DP of PMVS block is determined based on the ratio of the ring protons of styrene and vinyl protons ($H_{styrenic}/H_{vinyl} = 1.27$, shown in Fig. 2b in the main manuscript while DP of PS block is determined by the molecular weight values obtained from SEC for PS shown in Fig. 2a.

$$DP_{PS} = \frac{M_n\ from\ SEC}{Molar\ mass\ of\ styrene} = \frac{18600}{104.15} \approx 178$$

$$DP_{PMVS} = DP_{PS} \times \frac{H_{vinyl}}{H_{styrenic}} \times \frac{number\ of\ H_{styrenic}\ in\ each\ unit\ of\ PS\ block}{number\ of\ H_{vinyls}\ in\ each\ unit\ of\ PMVS\ block}$$

$$DP_{PMVS} = 178 \times \frac{1}{1.27} \times \frac{5}{3} \approx 234$$

Calculation of the graft number for bottlebrush block:

By knowing the grafting density and also the DP of PMVS block, the graft number is calculated as follows:

$$Graft\ number = grafting\ density \times DP_{PMVS} = 0.24 \times 234 \approx 56$$

2 Preliminary synthesis of PS-*b*-bbPDMS diblock

2.1 Structural parameters

PS-*b*-bbPDMS diblock copolymer features multiple topological parameters including the degrees of polymerization of linear PS block (N_L), bottlebrush backbone (N_{bb}), grafted side chains (N_{sc}), and the grafting density (Gr), as shown schematically in Figure S1. These structural parameters are independently tunable but collectively affect the self-assembly and mechanical properties. In this study a library of samples are prepared by altering two of these parameters, i.e. N_{sc} and Gr, while keeping other parameters constant in order to achieve a target viscoelastic behavior.

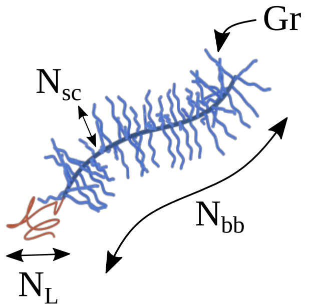

Figure S1: Schematic representation of the structural parameters of PS-*b*-bbPDMS diblock. N_L: degrees of polymerization of linear PS block, N_{bb}: degrees of polymerization of bottlebrush backbone, N_{sc}: degrees of polymerization of grafted side chains, and Gr: grafting density.

2.2 Synthesis of precursors

Monohydride-terminated polydimethylsiloxane (PDMS-H) with three different degree of polymerization are synthesized through the same synthesis protocol mentioned in the main manuscript by only changing the monomer to initiator ratio. ^{1}H NMR spectra of the synthesized PDMS-H with N_{sc} of 15, 33, and 45 are represented in Figure S2. PS-*b*-PMVS diblock copolymer is also separately synthesized through the same synthesis route as described in the main manuscript, leading to a diblock with N_L of 115 and N_{bb} of 148. ^{1}H NMR spectrum and the SEC curves of the diblock is shown in Figure S3.

2.3 Grafting step

Prepared PDMS-H side chains with three different lengths and the PS-*b*-PMVS diblock are dissolved in anhydrous toluene with specific weight ratios, aiming for three different grafting densities of 10, 20, and 30. The grafting reaction is carried out with the same route as described in the main manuscript but scale downed to a smaller batch size. The obtained PS-*b*-bbPDMS diblocks which differ in N_{sc} and Gr are labeled as $S_{Gr}^{N_{sc}}$. For instance, sample S_{10}^{15} represent sample with aimed grafting density of 10% with side chains length of 15. The grafting reaction are monitored by taking an aliqout from the reaction and running ^{1}H NMR measurement at days 3, 8, 12, 16 after the grafting has started. All these ^{1}H NMR spectra and the calculated grafting density are shown in Figures S4 - S6.

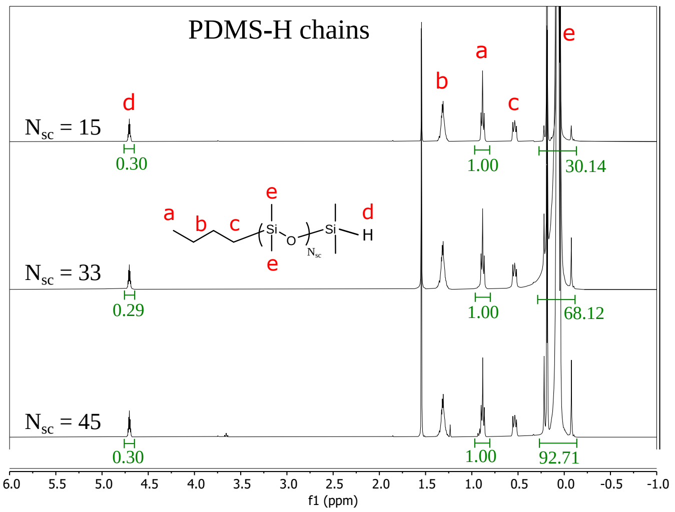

Figure S2: ^{1}H NMR spectra of the PDMS-H chains with different degree of polymerization polymerized until around 50% conversion. (400 MHz, $CDCl_3$, 25°C), $\delta = 0.07$ *ppm* (m, 3 H; $-Si-CH_3$), 0.53 *ppm* (t, 2 H, $-CH_2-$), 0.88 *ppm* (t, 3 H, $-CH_3$), 1.30 *ppm* (m, 4 H, $-CH_2-CH_2-$), 4.70 *ppm* (m, 1 H, $-Si-H$). $N_{sc} = \frac{e}{a\times 2} = \frac{30.14}{1\times 2} \approx 15$. $M_n = \frac{e}{a\times 2} \times \frac{molar\ mass\ of\ D_3}{3} = \frac{30.14}{1\times 2} \times \frac{222.46}{3} \approx 1120$ g.mol^{-1}.

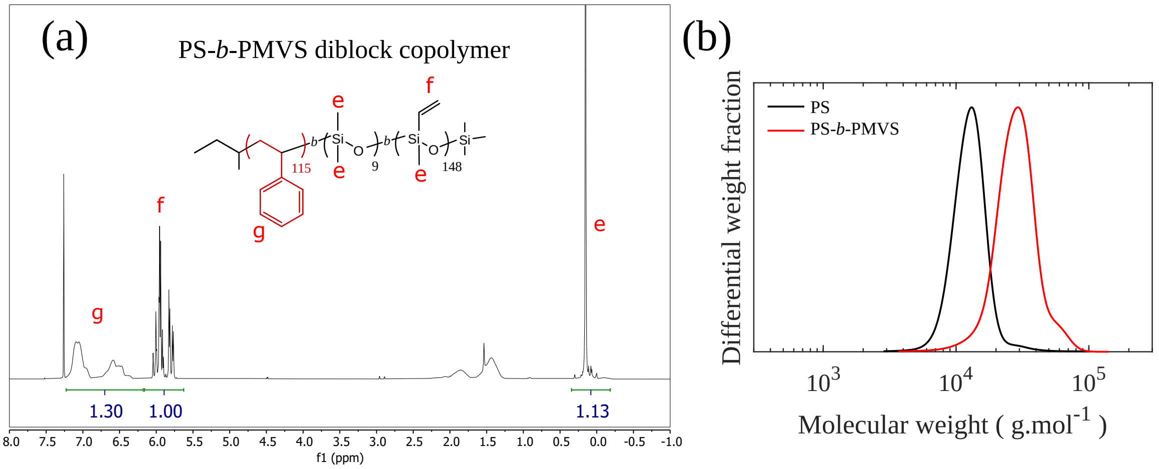

Figure S3: Molecular characterization of synthesized PS-*b*-PMVS diblock copolymer. **(a)** ^{1}H NMR spectrum. (400 MHz, $CDCl_3$, 25°C), $\delta = 0.13$ *ppm* (m, 3 H; $-Si-CH_3$), 5.72 – 6.05 *ppm* (m, 3 H, $-CH{=}CH_2$, 6.25 – 7.25 *ppm* (m, 5 H, $-C_6H_5$). **(b)** SEC curves for the PS block (—) with $M_n = 12000$, $M_w = 13200$ g.mol^{-1}, and Đ = 1.10, and PS-*b*-PMVS diblock (—) with $M_n = 26200$, $M_w = 30000$ g.mol^{-1}, and Đ = 1.14.

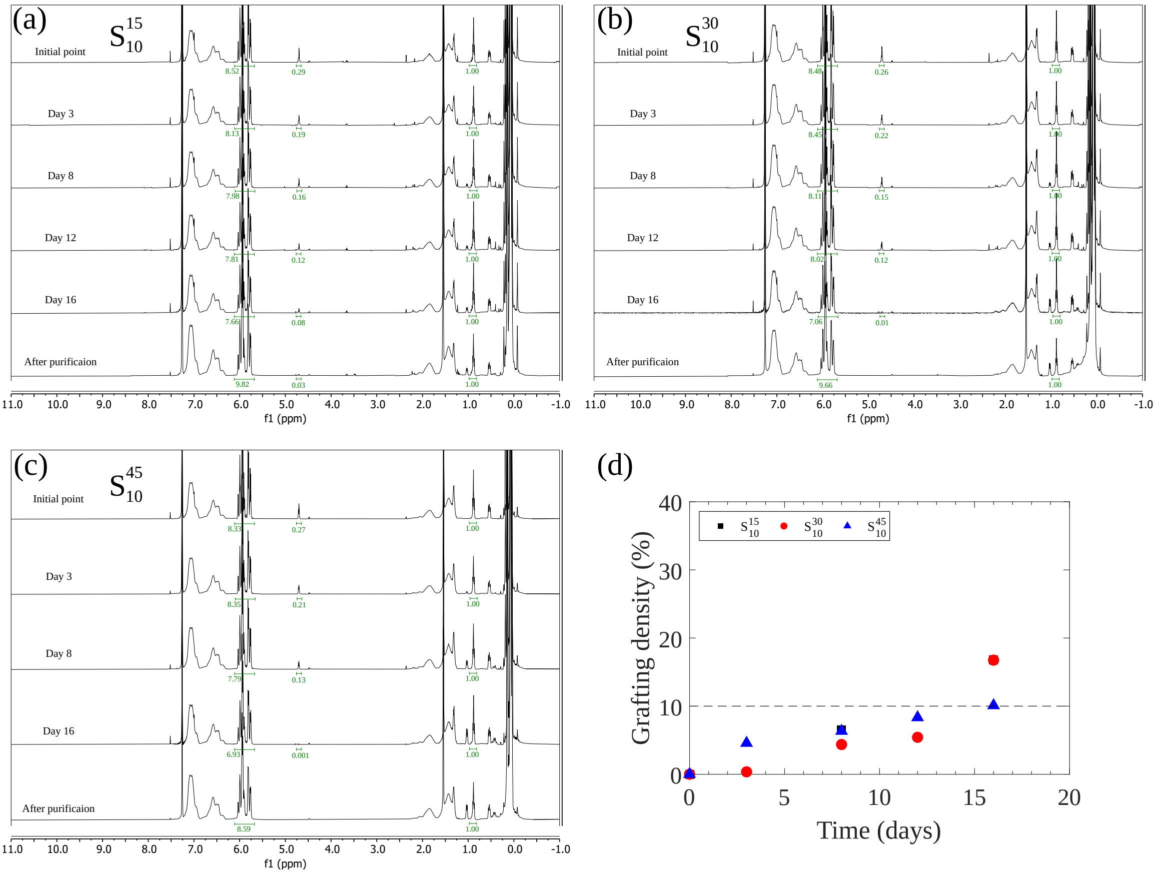

Figure S4: ^{1}H NMR spectra for synthesis of the PS-*b*-bbPDMS diblock copolymer for 10% aimed grafting density with side chains length of **(a)** N_{sc} = 15, **(b)** N_{sc} = 30, and **(c)** N_{sc} = 45 at different reaction stages and after purification. **(d)** Evolution of the grafting density during the grafting reaction calculated based on reduction of vinyl groups from ^{1}H NMR spectra. Dashed line represents the aimed grafting density considered to determine the stoichiometric mixing ratio between the side chains and the backbone.

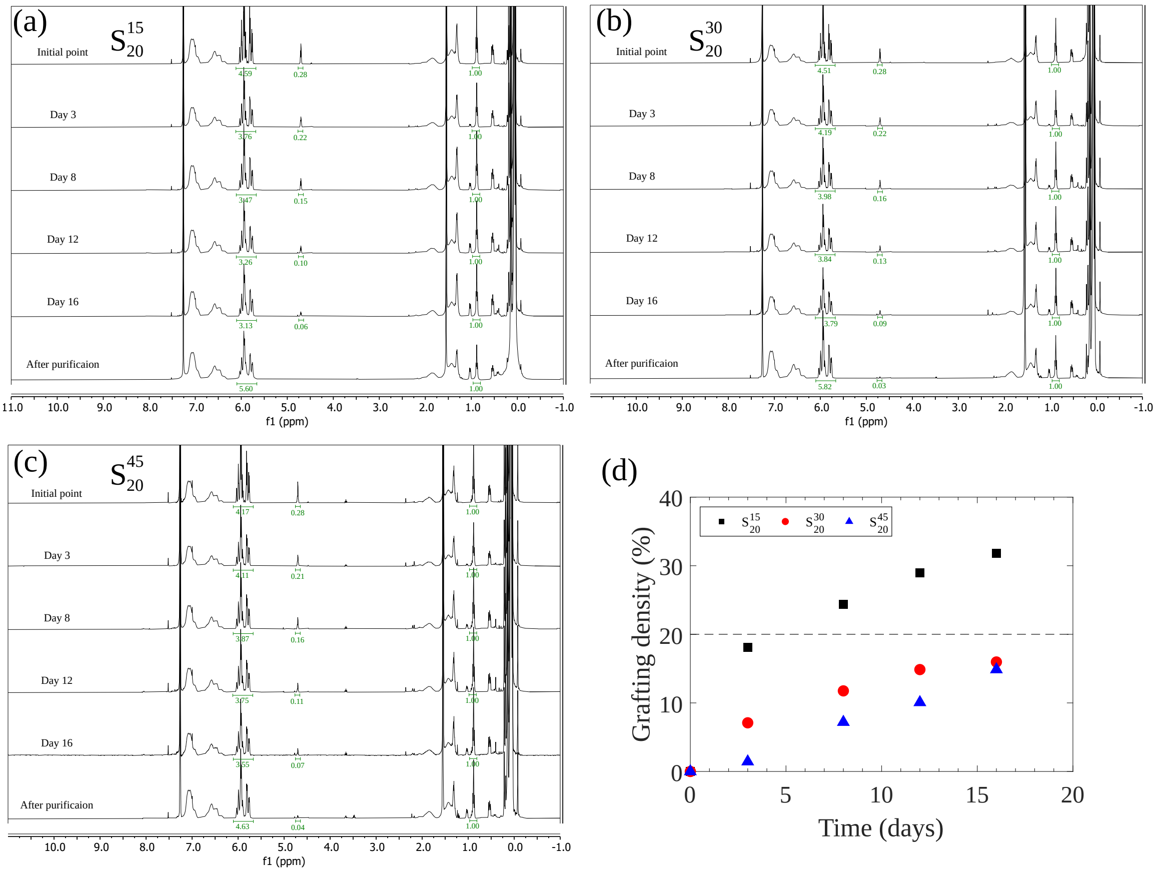

Figure S5: ^{1}H NMR spectra for synthesis of the PS-b-bbPDMS diblock copolymer for 20% aimed grafting density with side chains length of **(a)** $N_{sc} = 15$, **(b)** $N_{sc} = 30$, and **(c)** $N_{sc} = 45$ at different reaction stages and after purification. **(d)** Evolution of the grafting density during the grafting reaction calculated based on reduction of vinyl groups from ^{1}H NMR spectra. Dashed line represents the aimed grafting density considered to determine the stoichiometric mixing ratio between the side chains and the backbone.

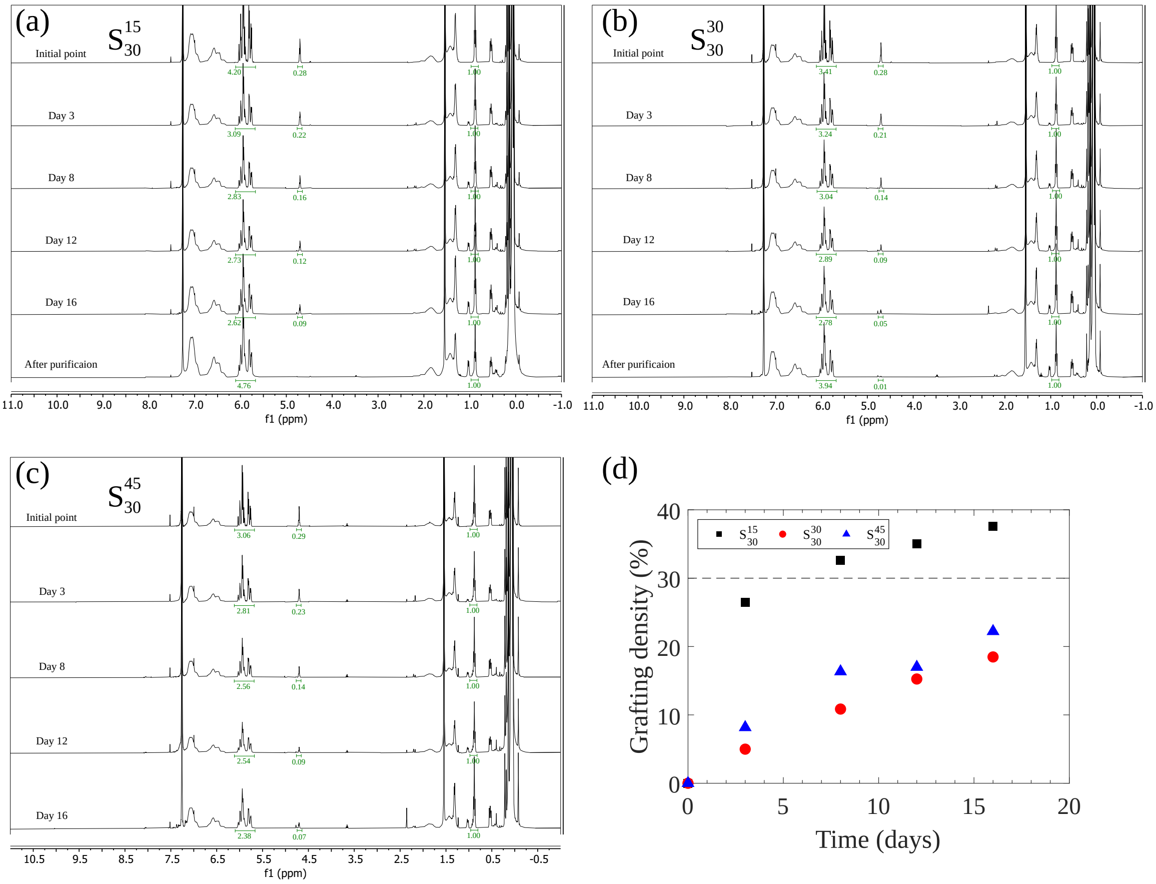

Figure S6: ^{1}H NMR spectra for synthesis of the PS-b-bbPDMS diblock copolymer for 30% aimed grafting density with side chains length of **(a)** $N_{sc} = 15$, **(b)** $N_{sc} = 30$, and **(c)** $N_{sc} = 45$ at different reaction stages and after purification. **(d)** Evolution of the grafting density during the grafting reaction calculated based on reduction of vinyl groups from ^{1}H NMR spectra. Dashed line represents the aimed grafting density considered to determine the stoichiometric mixing ratio between the side chains and the backbone.

2.4 Gravitational flow experiment

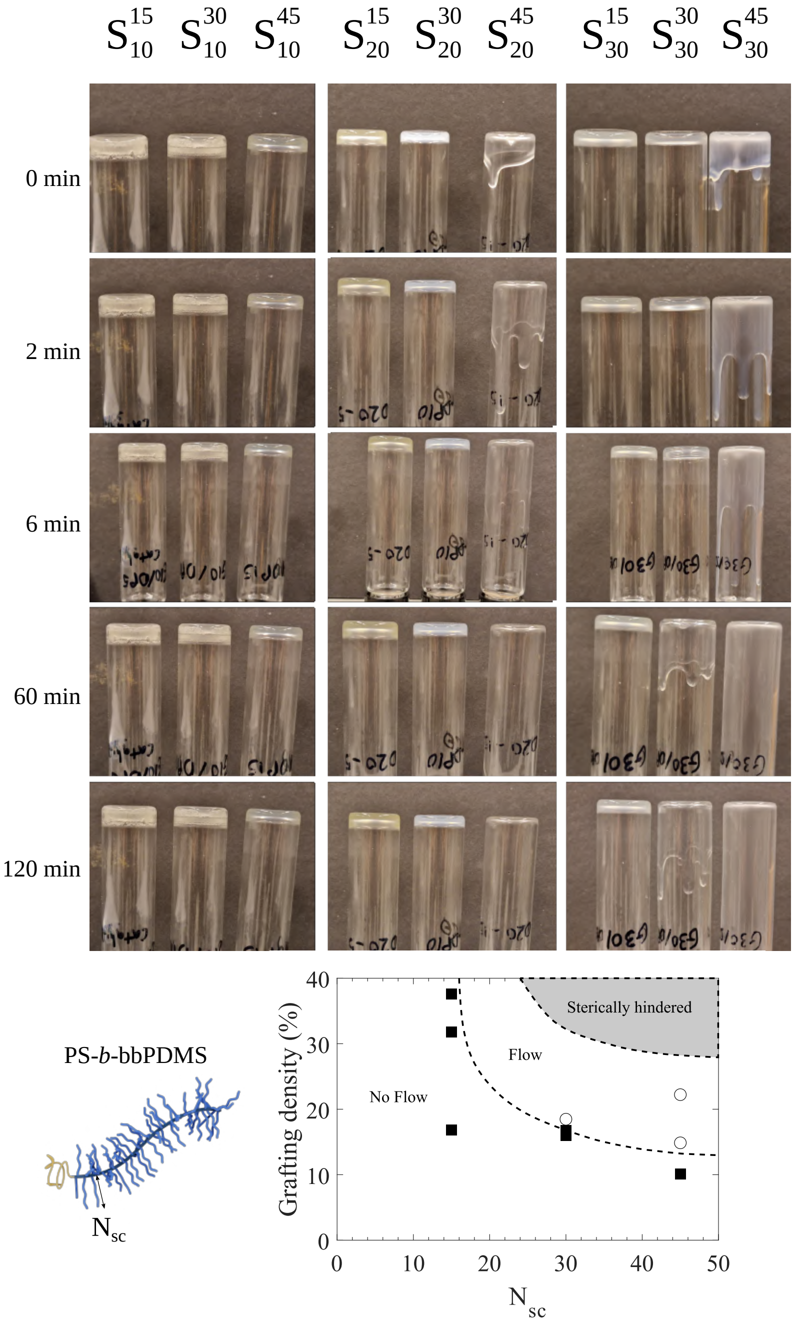

Figure S7: Pictures of vials containing the prepared samples in the upside down position at different times. Based on whether the sample flows under the gravitational force a qualitative phase diagram is plotted showing the grafting density as a function of side chain length. Solid symbols represent samples being intact after two hours of being upside-down, however, open symbols represent samples that flow either immediately or at any point within the two hours. Some combinations of grafting density and side chain length are impossible to experimentally synthesize through grafting onto approach due to the steric hindrance effects, shown as the grey area in this phase plot.

3 Transmission electron microscopy (TEM)

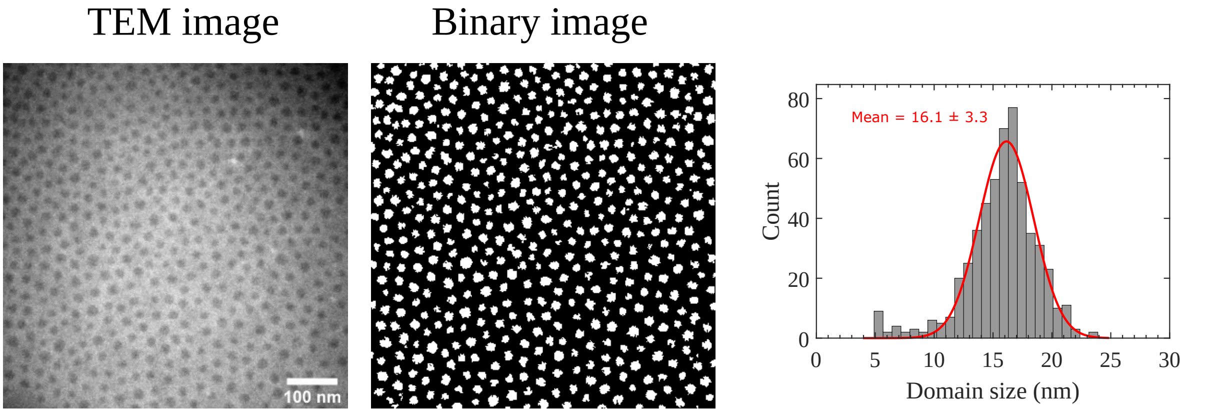

Figure S8: PS domain size distribution for the mixture of synthesized PS-*b*-bbPDMS diblock and PS-*b*-PDMS-*b*-PS (15-*b*-60-*b*-15 kg.mol^{-1}) triblock with 3:1 molar ratio obtained by analysis of TEM image. Using the binary image, shown above, a morphological calculation of equivalent disk area is used to define the phase separated domain size. Red line represents the Gaussian fit to the histogram of PS domain size.

4 Custom-made 3D printing setup

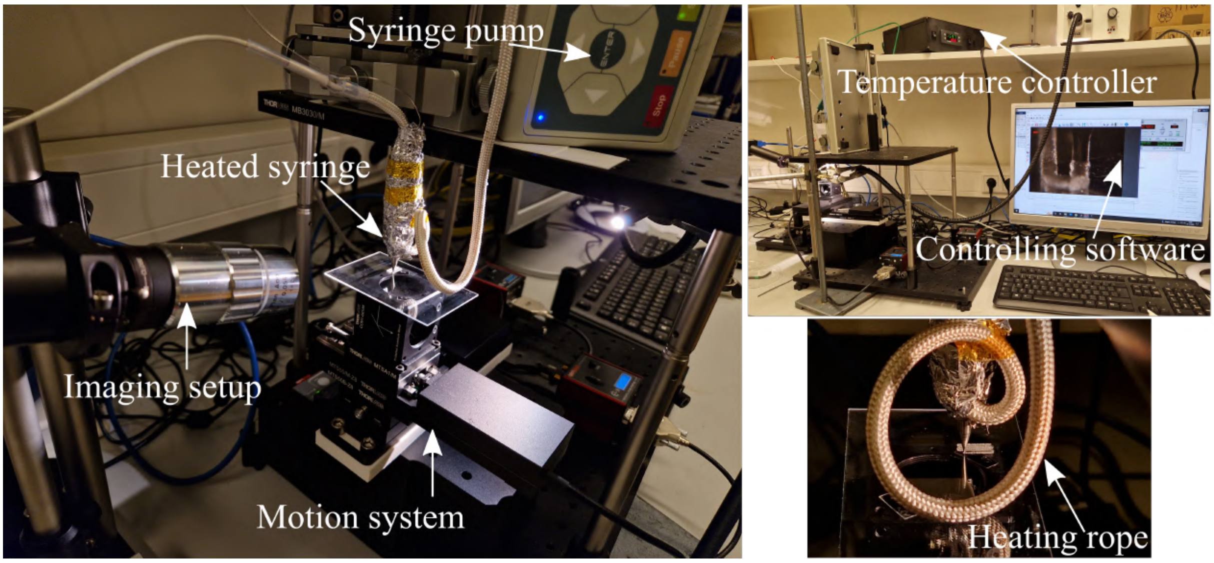

Figure S9: Custom-made 3D printing setup used for DIW printing of bottlebrush thermoplastic elastomer consisting mixture of PS-*b*-bbPDMS diblock and PS-*b*-PDMS-*b*-PS (15-*b*-60-*b*-15 kg.mol^{-1}) triblock copolymer with 3:1 molar ratio. The thermoplastic elastomer is loaded into a glass syringe which has been wrapped with a heating rope and sealed by aluminum foil. The Temperature of the material is read by a temperature sensor which has been taped on the outer side of the syringe and controlled by using a temperature controller. The rate of the injection and the motion trajectory are controlled through a MATLAB manuscript which converts G-Codes to motional commands for the translational stages.

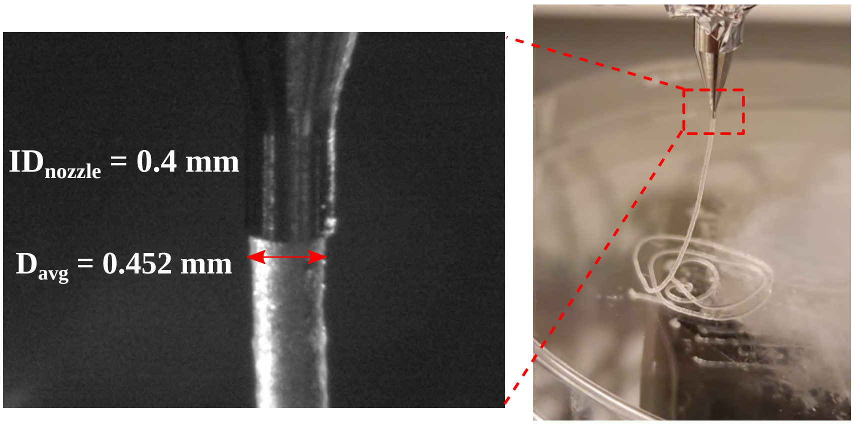

Figure S10: The die swell phenomenon as the extrudate comes out of the printing nozzle. The percentage of die swell is calculated as the ratio of the difference between diameter of extrudate (D_{avg}) and the inner diameter of the nozzle (ID_{nozzle}) over the inner diameter of the nozzle : Die swell (%) $= \frac{D_{avg} - ID_{nozzle}}{ID_{nozzle}} \times 100 = \frac{0.452-0.4}{0.4} \times 100 = 13$ %

Table S1: Overview of the 3D printing parameters

Printing parameters	3D Printed structures: Two-layer log-pile with different gap sizes		3D Printed structures: Four-layer log-pile structure	
	**First layer**	**Second layer**	**First layer**	**Other layers**
Flow rate (Q)	18 $\mu L/hr$	15 $\mu L/hr$	18 $\mu L/hr$	18 $\mu L/hr$
Nozzle translational speed (V_t)	0.04 mm/s	0.04 mm/s	0.04 mm/s	0.04 mm/s
Nozzle tip-surface distance (h)	0.3 mm	0.4 mm	0.25 mm	0.4 mm
Center-to-center distance between lines (L)	1, 1.5, 2, 2.25, 2.5 mm	2.5 mm	1 mm	1 mm
Nozzle temperature / print bed temperature	160°C / 20°C	160°C / 20°C	160°C / 20°C	160°C / 20°C
Nozzle inner diameter	0.4 mm	0.4 mm	0.4 mm	0.4 mm
